# Supplementary material for: Complexome profiling of the Chlamydomonas psb28 mutant reveals TEF5 as an early PSII assembly factor
Source: Plant Cell. 2025 Mar 17;37(6):koaf055. doi: 10.1093/plcell/koaf055 (PMC12164590; doi:10.1093/plcell/koaf055)
Supplement: koaf055_Supplementary_Data [file koaf055_supplementary_data.zip › psb28_interactive.zip!/plots/intensity/qualityPlots/peptides/GLC2A_Cre03.g190500.t1.2.html]

### Cre03.g190500.t1.2

Trivial name: GLC2A  
SeqCoverage [median]: 14.00  
Mass: 100.69 kDa  
  
 

| Sequence | MissedCleavage | Mass [Da] | wtIntensity | muIntensity | Score |
| --- | --- | --- | --- | --- | --- |
| AFFSGTQR | 0 | 912.45 | 0.00 | 632.74 | 69.20 |
| ATSLSLK | 0 | 718.42 | 0.00 | 709.53 | 81.78 |
| AVISDGVSGAK | 0 | 1002.53 | 516.43 | 0.00 | 76.83 |
| DNLHYGNVEHR | 0 | 1352.62 | 0.00 | 324.61 | 66.26 |
| GHAHLETQR | 0 | 1047.52 | 238.71 | 792.24 | 67.99 |
| GSQVDPENGDRPFVLSR | 1 | 1871.91 | 0.00 | 598.99 | 26.29 |
| GVPALQLNSR | 0 | 1053.59 | 345.09 | 896.49 | 67.99 |
| HTVALEAGPLSMAAAPDSTQALVLR | 0 | 2518.32 | 0.00 | 2170.42 | 114.04 |
| HVEPGVPDGTLTVPTTIER | 0 | 2017.05 | 580.68 | 3173.25 | 73.92 |
| VVFLGLPAAK | 0 | 1013.63 | 1940.94 | 0.00 | 77.67 |
|  |  |  | 3621.8 | 9298.3 |  |
